# The Relationship between Authoritarian Leadership and Employees’ Deviant Workplace Behaviors: The Mediating Effects of Psychological Contract Violation and Organizational Cynicism

**DOI:** 10.3389/fpsyg.2017.00732

**Published:** 2017-05-09

**Authors:** Hongyan Jiang, Yang Chen, Peizhen Sun, Jun Yang

**Affiliations:** ^1^School of Management, China University of Mining and TechnologyXuzhou, China; ^2^Department of Psychology, Jiangsu Normal UniversityXuzhou, China

**Keywords:** authoritarian leadership, deviant workplace behaviors, psychological contract violation, organizational cynicism, mediating effects

## Abstract

This study investigated the relationship between authoritarian leadership and employees’ deviant workplace behaviors (DWB), as well as the mediating effects of psychological contract violation and organizational cynicism. A cross-sectional survey was conducted among 391 manufacturing workers in a northern city of China. Structural equation modeling was performed to test the theory-driven models. The results showed that the relationship between authoritarian leadership and employees’ DWB was mediated by organizational cynicism. Moreover, this relationship was also sequentially mediated by psychological contract violation and organizational cynicism. This research unveiled psychological contract violation and organizational cynicism as underlying mechanism that explained the link between authoritarian leadership and employees’ DWB.

## Introduction

Deviant workplace behaviors (DWB) are increasing dramatically, which has recently drawn extensive attention among both academicians and practitioners. Robinson and Bennett (1995) have described DWB as any voluntary acts that violate organizational norms, and thus threaten the well-being of an organization, its members, or both. A report by the US Chamber of Commerce estimates that 75% of employees steal at least once (Shulman, 2005). Moreover, DWB can be linked with adverse aspects of individuals, groups and organizations (Alias et al., 2013). For instance, unauthorized web surfing (gambling) during working hours has been estimated to cost up to £300 million loss in productivity yearly (Taylor, 2007). The majority of previous research has concentrated on the relationship between employees’ personality traits (e.g., O’Neill and Hastings, 2011; Zhang et al., 2015) and their DWB. However, very little empirical research has been done on the link between leadership style and employees’ DWB.

Leaders represent their organizations, and their actions are often related to followers’ behaviors (Aquino et al., 1999). Leadership has been conceptualized as the process of influencing the activities of an organized group towards the task accomplishment (Chemers, 1997). Among the various leadership styles, authoritarian leadership is one of the most prevalent in Chinese settings (Farh and Cheng, 2000), due to its fit with traditional cultures. Thus, authoritarian leadership has been chosen as our interested leadership construct. Authoritarian leadership is originally defined by Cheng et al. (2004) as one element of paternalistic leadership. They argue that authoritarian leadership can be conceptualized as leaders’ behaviors that assert absolute authority and control over subordinates and demand unconditional obedience. High leadership authority means low sharing of power and information with followers, as well as strong control over followers’ behaviors.

Bass (1990) has classified the main styles of leadership into transactional, transformational, empowering, and authoritarian. The first three styles could be summarized as egalitarian leadership, which stresses the notion of equal distribution of power in the community or group (Flood et al., 2000). In contrast to egalitarian leadership, authoritarian leadership places emphasis on the asymmetric power between leaders and followers, which allows leaders to put personal dominance and control over followers (Tsui et al., 2004). Moreover, authoritarian leadership differs from some other types of leadership, such as abusive supervision, which is described as supervisors’ sustained display of non-physical hostility against their subordinates (Tepper, 2000); authentic leadership, which is defined as leaders’ behaviors aiming at promoting positive psychological capacities of employees (Walumbwa et al., 2008). Compared with the above two leadership styles, the core of authoritarian leadership is asserting complete control over subordinates.

Previous research has identified some personality variables which may relate to authoritarian leadership, such as supervisors’ machiavellianism (Kiazad et al., 2010), need for personalized power (Aryee et al., 2007) and narcissism (Conger and Kanungo, 1998). Another stream of research has found that authoritarian leadership is associated with employees’ attitude, affect and behaviors, such as job dissatisfaction (Shaw, 1955), negative emotions towards supervisors (Farh et al., 2006), and extra-role behaviors (Chen et al., 2014). Although authoritarian leadership may also relate to employees’ DWB, scant attempts have been made to systematically explore the underlying mechanism behind the relationship between authoritarian leadership and employees’ DWB.

Social Exchange Theory states that the basic nature of human behaviors is a subjective interaction with others, and the development of interpersonal relationships is in accordance with the norm of reciprocity (Gouldner, 1960; Blau, 1964). According to this theory, leadership behaviors may shape the exchange relationship between leaders and followers, which might be associated with followers’ workplace behaviors. Moreover, psychological contract violation could be considered as subordinates’ unfulfilled expectation of the social exchange relationship (Brown and Moshavi, 2002). In addition, organizational cynicism is “a negative attitude toward one’s employing organization” (Dean et al., 1998). Employees with high level of organizational cynicism hold that the organization lacks integrity and that leader’s decisions are made with a self-interested drive (Andersson, 1996; Neves, 2012). From the Social Exchange Theory perspective, it is thus reasonable to infer that both psychological contract violation and organizational cynicism, which could reflect the quality of social relation between leaders and followers, may mediate the link between authoritarian leadership and employees’ DWB. Therefore, drawing on Social Exchange Theory, the current research is to investigate the relationship between authoritarian leadership and DWB. More importantly, we attempt to explain why this relationship occurs by providing important explanatory mechanism. To this end, psychological contract violation and organizational cynicism are introduced as mediators that account for the authoritarian leadership-DWB relationship.

## Theoretical Background and Hypotheses

### Authoritarian Leadership and Employees’ DWB

Authoritarian leadership refers to a leader’s behaviors of implementing strong control over subordinates and requiring their unconditional obedience (Cheng et al., 2004). The main characteristic of authoritarian leadership is absolute dominance of the leaders. Authoritarian leaders are inclined to exert control by issuing rules and threatening punishment for disobedience (Aryee et al., 2007). They often apply strict discipline to subordinates’ work and exhibit their authority on decision making (Wang et al., 2013). When leaders implement their followers with an authoritarian approach, subordinates are demanded to comply with leaders’ requests without dissent and subordinates may experience negative emotions towards leaders (Farh et al., 2006). Prior research has shown that authoritarian leadership is linked with employees’ job dissatisfaction (Shaw, 1955). Furthermore, when employees are dissatisfied with their job, they may exhibit DWB like absenteeism, low performance and violence (Mount et al., 2006). Thus, it is inferred that authoritarian leadership is positively linked with employees’ DWB.

Based on Social Exchange Theory, all human behaviors are based on the reciprocal benefits in the social relationship, and the benefits exchanged are indicative of mutual support and investment in that relationship (Gouldner, 1960; Blau, 1964; Neves and Caetano, 2006). According to the norm of reciprocity (Gouldner, 1960), subordinates’ attitude and behaviors are associated with leadership behaviors. When subordinates receive support or administration authority from their leaders, they are inclined to reciprocate with positive job attitude and performance. On the contrary, when subordinates are subjected to threats or intimidation from authoritarian leaders (Kiazad et al., 2010), they tend to reciprocate with negative reactions, such as DWB. Taken together, it is possible that employees under authoritarian leadership are more likely to exhibit DWB. Therefore, this study proposes the following hypothesis:

H1: Authoritarian leadership is positively related to employees’ DWB.

### Mediating Role of Psychological Contract Violation

Psychological contract is an individual’s belief regarding the terms of an agreement between the individual and organization (Levinson et al., 1972; Kotter, 1973). The critical element of this concept lies in the reciprocal obligations. For instance, subordinates expect to receive rewards in exchange for their commitment and contribution to the organization. Psychological contract violation is defined as subordinates’ perception that the organization has failed to fulfill its obligations or promises (Morrison and Robinson, 1997). When the organization is unable to meet subordinates’ expectation, the psychological contract violation occurs. Authoritarian leaders often disregard followers’ suggestions and discount their contributions (Aryee et al., 2007), which makes them feel disrespected and their expectation unmet. Therefore, employees under authoritarian leadership are inclined to experience psychological contract violation.

Moreover, authoritarian leaders often control and command subordinates mainly via threats and intimidation (Kiazad et al., 2010), which could be related to employees’ negative emotions, such as anger and fear (Farh et al., 2006). These negative emotions are associated with psychological contract violation (Ortony et al., 1988; Morrison and Robinson, 1997). Thus, employees under authoritarian leadership may feel angry and fearful towards the organization and consider whether to maintain or end this organization-member relationship, and then the psychological contract is likely to be violated. Based on these previous studies, we propose the following hypothesis:

H2a: Authoritarian leadership is positively linked with psychological contract violation.

A growing literature has suggested that psychological contract violation is linked with employees’ DWB (e.g., Restubog et al., 2007; Bordia et al., 2008). General Strain Theory (Agnew, 1992, 2006) has posited that strain occurs when negative social relationship is developed, and individuals who experience high level of accumulated strain are inclined to engage in deviant behaviors. As we know, when psychological contract is violated, employees perceive relatively lower quality relationship with their organization. Drawing from the General Strain Theory, lower quality relationship would force employees into a high-strain situation. Taking this into consideration, exhibiting DWB would be considered as a reaction to the strain. Therefore, employees with higher level of strain are more likely to show DWB (Alias et al., 2013). Based on both theoretical basis and empirical findings, it is possible that psychological contract violation is positively associated with employees’ DWB. Thus, this study puts forward the following hypothesis:

H2b: Psychological contract violation is positively related to employees’ DWB.

The foundation of the organization-member relationship is psychological contract, which is comprised of beliefs about reciprocal obligations in this social relationship (Rousseau, 1989). When subordinates perceive that leaders fail to fulfill obligations or promises, the psychological contract violation occurs (Turnley et al., 2003). Accordingly, the research of psychological contract violation has generally taken Social Exchange Theory to understand its relationship with employees’ behaviors (Chen et al., 2004). As suggested in Social Exchange Theory (Blau, 1964; Lorinkova and Perry, 2014), leadership behaviors could shape the mutual relationship between leaders and subordinates, which may be linked with subordinates’ behaviors. Based on this theory, authoritarian leadership which makes employees’ social exchange expectation unfulfilled, may be related to employees’ higher psychological contract violation, and then employees are more likely to exert DWB. Taking these theoretical arguments and aforementioned hypotheses (H1, H2a, and H2b) into consideration, it is inferred that psychological contract violation may act as a mediator between authoritarian leadership and employees’ DWB. We propose the following hypothesis:

H2c: Psychological contract violation mediates the link between authoritarian leadership and employees’ DWB.

### Organizational Cynicism as a Mediator

Organizational cynicism is defined as “a negative attitude toward one’s employing organization, comprising three dimensions: (1) a belief that the organization lacks integrity; (2) negative affect toward the organization; and (3) tendency of showing critical behaviors towards the organization” (Dean et al., 1998). Organizational cynicism is characterized by frustration, hopelessness, contempt toward organization and lack of trust in organization (Andersson, 1996). The main reason for the link between authoritarian leadership and organizational cynicism lies in the variation of perceived organizational support. It is well acknowledged that authoritarian leaders emphasize personal dominance and control over subordinates, and they habitually get things done in their own ways (Tsui et al., 2004). Furthermore, authoritarian leaders often disregard the interests and perspectives of employees (Chan et al., 2013). Since leadership behavior operates as an important indicator of the extent of support provided by the organization (Levinson, 1965), subordinates under authoritarian leadership may feel that they get less support from the organization. Moreover, this reduction of perceived organizational support could be linked with followers’ cynical attitudes towards the organization (Leiter and Harvie, 1997; Treadway et al., 2004). Thus, it is plausible that authoritarian leadership is positively related to organizational cynicism. This study proposes the following hypothesis:

H3a: Authoritarian leadership is positively linked with organizational cynicism.

It has been demonstrated that organizational cynicism is positively linked with employees’ DWB (e.g., James, 2005; Evans et al., 2010). Organizational cynics believe that leader is concerned only with his own self-interest, and they usually feel frustrated and being treated unfairly (Andersson, 1996). Moreover, frustration (Spector, 1997) and perceived injustice (Greenberg and Alge, 1998) are positively associated with employees’ DWB. Thus, employees with higher organizational cynicism are more likely to show DWB. Taken together, it is reasonable to infer a positive link between organizational cynicism and employees’ DWB. Therefore, this study proposes the following hypothesis:

H3b: Organizational cynicism is positively related to employees’ DWB.

According to the Social Exchange Theory (Gouldner, 1960; Blau, 1964), the behaviors of leaders, the key agents of the organization, would shape subordinates’ attitude and behaviors towards the organization. From this theory, authoritarian leadership which stresses on leaders’ dominate control over subordinates via threats and intimidation (Kiazad et al., 2010), would make subordinates feel uneasy, oppressed and generate distrust in organization (Wu et al., 2012). This sense of distrust is the main characteristic of organizational cynicism (Kanter and Mirvis, 1989; Andersson, 1996). Then, subordinates with high level of organizational cynicism may experience frustration and contempt towards organization (Andersson, 1996) and tend to engage DWB. Taking theoretical arguments and these above hypotheses (H1, H3a, and H3b) into consideration, authoritarian leaders who are arbitrary and of low trustworthiness could make employees to be cynical towards the organization, which may be related to employees’ DWB. Thus, it is plausible that organizational cynicism might account for the relationship between authoritarian leadership and employees’ DWB. The following hypothesis is tested:

H3c: Organizational cynicism mediates the link between authoritarian leadership and employees’ DWB.

Many studies have shown that psychological contract violation could be associated with organizational cynicism (e.g., Andersson, 1996; Dean et al., 1998; Chrobot-Mason, 2003; Johnson and O’Leary-Kelly, 2003). As we know, psychological contract violation involves employees’ perception that their employing organization fails to fulfill promised obligations or duties (Morrison and Robinson, 1997). Furthermore, this negative perception (i.e., psychological contract violation) could make employees feel distrust towards their organization, and then the cynical attitude towards organization may be strengthened (Robinson et al., 1994). On the basis of these studies, we propose the following hypothesis:

H4: Psychological contract violation is positively related to organizational cynicism.

**FIGURE 1:**
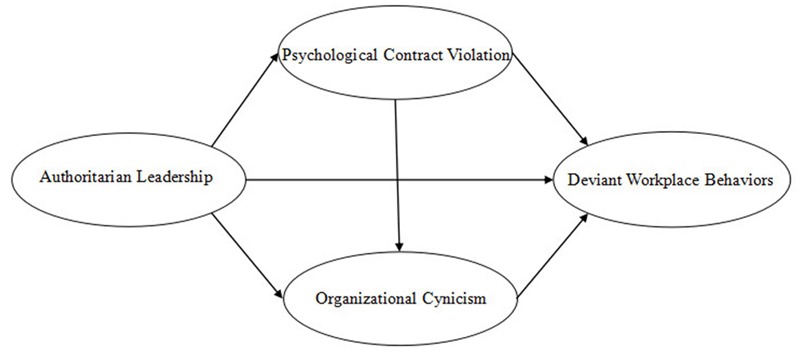
**Hypothetical model**.

To sum up, a set of hypotheses have been derived from existing theory and prior research. Then several multivariate models will be constructed to test these hypotheses, specifically the proposed mediating roles of psychological contract violation and organizational cynicism between authoritarian leadership and DWB. Based on these analyses mentioned above, the present study puts forward the following hypothetical model shown in **Figure 1**.

## Materials and Methods

### Participants and Procedures

The sample consisted of 391 workers from five manufacturing enterprises in a northern city of China. We collected data from full-time employees who had worked together and frequently interacted with their supervisors. At first, a total of 470 questionnaires were distributed, and 453 questionnaires were returned for an overall return rate of 96.4%. Two surveys were not included due to incomplete or illegible responses. Additionally, the participants who indicated that they did not have a supervisor had been excluded, because they were unable to provide meaningful ratings on the focal variable, perceived authoritarian leadership. Finally, 391 (83.2%) valid questionnaire were received.

The jobs held by these employees varied widely, including repairman, quality inspectors, operator, material manager, and other manufacture related activities. The respondents were mainly men (*n* = 271, 69.3%), and they are mostly between the ages of 26 and 45, which make up 60.4% of the total. The majority of the participants were junior college (vocational education, 36.5%) and undergraduate college (undergraduate education, 42.0%). Moreover, 60.2% of the respondents possessed 3–5 years’ work experience. In addition, 10.6% of the participants had a low- or mid-level leadership position (7.4% first-line manager, 3.2% department middle management staff).

From January to February 2015, we contacted with several manufacturing enterprises and asked them to participate in this investigation. After getting approval, the managers of each enterprise introduced the human resource department staffs to us. We told them the purpose of this survey, proper ways of collecting data in addition to the detailed precautions in the survey. At the beginning of the investigation, we introduced the voluntary nature of this survey and assured anonymity and confidentiality to the participants. To express our appreciation, participants were given a $5 gift certificate to a local store as long as they completed the questionnaire. This study was part of a larger research project on leadership behaviors, for which the first author had received ethical clearance from the university ethical review process.

### Measures

A 5-point Likert scale ranging from strongly disagree (1) to strongly agree (5), was used to measure the participants’ responses for each item. The scales used to measure each variable were adapted from relevant prior research. All scales items were translated into Chinese by professional translators following a double blind back-translation procedure (Schaffer and Riordan, 2003) to ensure semantic equivalence with the original English wording. Cronbach’s alpha was calculated for each scale.

### Authoritarian Leadership

Authoritarian leadership was measured by the 9-item version of the Authoritarian Leadership Questionnaire by Farh and Cheng (2000). Sample items were “My supervisor asks me to obey his/her instructions completely”, “My supervisor always has the last say in the meeting”, and “My supervisor scolds us when we cannot accomplish our task”. Cronbach’s alpha of the scale was 0.87.

### Psychological Contract Violation

Psychological contract violation was measured by the Psychological Contract Violation Questionnaire (Robinson and Morrison, 2000). Sample items include “I feel betrayed by my organization”, “I feel that my organization has violated the contract between us”. Cronbach’s alpha was 0.90 for this scale.

### Organizational Cynicism

We used the Organizational Cynicism Questionnaire developed by Dean et al. (1998) to measure organizational cynicism. Examples of statements are “Company policies, goals and practices are often inconsistent”, “I feel angry when I think of the company”, “I often laugh at the company’s slogan and actions”. The scale consisted of three dimensions: cynicism faith (α = 0.92), cynicism emotion (α = 0.93) and cynicism behaviors (α = 0.84). Cronbach’s alpha of the total scale was 0.94.

### Deviant Workplace Behaviors

Deviant Workplace Behaviors Questionnaire (Bennett and Robinson, 2000) was adopted to measure DWB. It was a multidimensional construct including two portions: (a) 7 items for interpersonal deviance (α = 0.91), (b) 12 items for organizational deviance (α = 0.93). The example items were “Acted rudely toward someone at work”, “Dragged out work in order to get overtime”. Cronbach’s alpha was 0.95 for the total scale.

## Results

### Description

**Table 1** shows the mean, standard deviation, and correlations for each of the constructs. It was found that authoritarian leadership was significantly and positively correlated with employees’ DWB (*r* = 0.23, *p* < 0.01), psychological contract violation (*r* = 0.33, *p* < 0.01) and organizational cynicism (*r* = 0.38, *p* < 0.01). These results provided initial support for H1, H2a, and H3a, respectively. Psychological contract violation was significantly and positively correlated with DWB (*r* = 0.60, *p* < 0.01) which provided initial support for H2b. Organizational cynicism was significantly and positively correlated with DWB (*r* = 0.37, *p* < 0.01) and psychological contract violation (*r* = 0.59, *p* < 0.01) which provided initial support for H3b and H4.

**Table 1:** Descriptive statistics and correlations among all variables.

	1	2	3	4
(1) Authoritarian leadership	(0.87)			
(2) Organizational cynicism	0.38^∗∗^	(0.94)		
(3) Psychological contract violation	0.33^∗∗^	0.59^∗∗^	(0.90)	
(4) Deviant workplace behaviors	0.23^∗∗^	0.37^∗∗^	0.60^∗∗^	(0.95)
Mean	3.36	2.10	2.03	3.03
SD	0.69	0.52	0.62	0.37

*Cronbach’s alpha coefficients are in parentheses on the diagonal.*

*^∗∗^*p* < 0.01.*

### Measurement Model Testing

The measurement model consisted of four latent factors (authoritarian leadership, psychological contract violation, organizational cynicism and DWB) and eleven observed indicators. The observed indicators were formed by the method of item parceling (i.e., aggregating individual items into several parcels). Compared with item-level data, aggregate-level data has several advantages, such as higher communality, higher ratio of common-to-unique factor variance, and lower random error (Matsunaga, 2008). The goodness of fit of the model was evaluated using the following indices (Kline, 2005): (a) chi-square statistics; (b) root-mean-square error of approximation (RMSEA): best if below.08; (c) goodness-of-fit index (GFI), normed fit index (NFI), comparative fit index (CFI): best if above 0.90.

We conducted a confirmatory factor analysis (CFA) with maximum likelihood estimation using AMOS 21.0 to examine whether employees’ scores on their self-report measures (i.e., authoritarian leadership, psychological contract violation, organizational cynicism and DWB) captured distinctive constructs. We compared the fitness between a one-factor model (all observed indicators loaded on one factor), two-factor model (authoritarian leadership and psychological contract violation on one factor, organizational cynicism and DWB on the other), three-factor model (authoritarian leadership and psychological contract violation on one factor, organizational cynicism and DWB as separate factors) and four-factor model (authoritarian leadership, psychological contract violation, organizational cynicism and DWB as separate factors). The results (see **Table 2**) indicated that the four-factor model fitted the data better than other models.

**Table 2:** Fit indices for measurement models.

Structure	χ^2^	*df*	GFI	NFI	CFI	RMSEA
1-factor	355.52	44	0.85	0.83	0.83	0.085
2-factor	333.25	43	0.87	0.86	0.85	0.077
3-factor	291.11	41	0.88	0.90	0.89	0.074
4-factor	144.02	38	0.93	0.92	0.92	0.069
5-factor	130.95	27	0.93	0.92	0.93	0.065

*N = 391. 1-factor: AL + PCV + OC + DWB; 2-factor: AL + PCV, OC + DWB; 3-factor: AL + PCV, OC, DWB; 4-factor: AL, PCV, OC, DWB; 5-factor: AL, PCV, OC, DWB, CMV. AL, authoritarian leadership; OC, organizational cynicism; PCV, psychological contract violation; DWB, deviant workplace behaviors; CMV, common method variance.*

In order to determine whether common method variance was problematic, we employed Harman’s single-factor test (Harman, 1976) to test whether the majority of the variance could be accounted for by one general factor. The results showed that the first factor accounted for only 29.46% of the variance, less than half, and this finding could be accepted (Harris et al., 2013). Furthermore, common method variance was tested by using the CFA marker technique (Podsakoff et al., 2012). We performed a CFA (5-factor model) in which a common method factor was added to 4-factor model. The results indicated the inclusion of the common method variance in 5-factor model did not improve the overall model fit of 4-factor model significantly (**Table 2**). Thus, it was determined that the common method bias was not a problem in this study.

### Structure Model Testing

**FIGURE 2:**
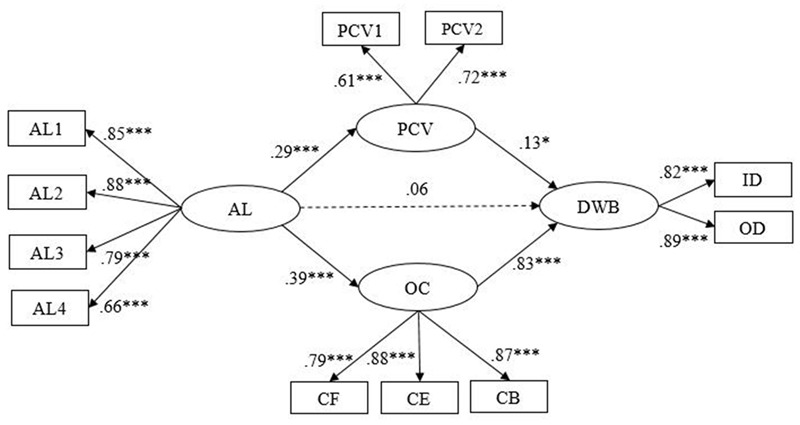
**Partial-mediated model (Model 1).** AL1–AL4 are four parcels of authoritarian leadership (AL1–AL3 aggregates of two items and AL4 was three items from the Authoritarian Leadership Questionnaire); PCV, psychological contract violation; OC, organizational cynicism; DWB, deviant workplace behaviors; PCV1 aggregates of two items and PCV2 was two items from Psychological Contract Violation Questionnaire; CF, cynicism faith; CB, cynicism behavior; CE, cynicism emotion; CF, CB, and CE are three dimensions of the Organizational Cynicism Questionnaire; ID, interpersonal deviation; OD, organizational deviation; ID and OD are two dimensions of the Deviant Workplace Behaviors Questionnaire. *^∗^p* < 0.05; ^∗^*^∗^p* < 0.01; *^∗∗∗^p* < 0.001.

**FIGURE 3:**
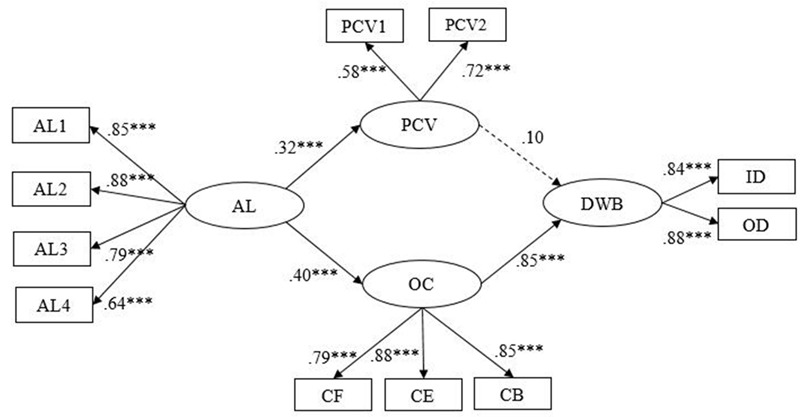
**Fully mediated model (Model 2).** AL1–AL4 are four parcels of authoritarian leadership (AL1–AL3 aggregates of two items and AL4 was three items from the Authoritarian Leadership Questionnaire); PCV, psychological contract violation; OC, organizational cynicism; DWB, deviant workplace behaviors; PCV1 aggregates of two items and PCV2 was two items from Psychological Contract Violation Questionnaire; CF, cynicism faith; CB, cynicism behavior; CE, cynicism emotion; CF, CB, and CE are three dimensions of the Organizational Cynicism Questionnaire; ID, interpersonal deviation; OD, organizational deviation; ED and OD are two dimensions of the Deviant Workplace Behaviors Questionnaire. *^∗^p* < 0.05; ^∗^*^∗^p* < 0.01; *^∗∗∗^p <* 0.001.

Structural equation modeling (SEM) was used to test our Hypotheses 1–4 and to assess the appropriateness and fit of our proposed theoretical model. First, we built a partial-mediated model (Model 1). The results showed that Model 1 did not fit the data well (see **Table 3**), and the path from authoritarian leadership to employees’ DWB was not significant (β = 0.06, *p* > 0.05) (see **Figure 2**). Then we deleted the non-significant path based on the Model 1 and built the fully mediated model (Model 2) (see **Figure 3**). The results indicated that Model 2 fitted well to the data (see **Table 3**), but the path from psychological contract violation to employees’ DWB was non-significant (β = 0.10, *p* > 0.05). The chi-square difference between Model 1 and Model 2 reached the significant level (Δχ^2^ (1) = 60.80, *p* < 0.001), which suggested that Model 2 fitted better than Model 1 (see **Table 3**).

**Table 3:** Comparison of the structural models.

Model	χ^2^	*df*	GFI	NFI	CFI	RMSEA
M1 (Partially mediated model)	242.52	39	0.86	0.84	0.89	0.095
M2 (Fully mediated model)	181.72	40	0.92	0.91	0.94	0.068
M3 (The final model)	135.88	39	0.95	0.95	0.96	0.057

*GFI, goodness-of-fit index; NFI, normed fit index; CFI, comparative fit index; RMSEA, root mean square error of approximation.*

**FIGURE 4:**
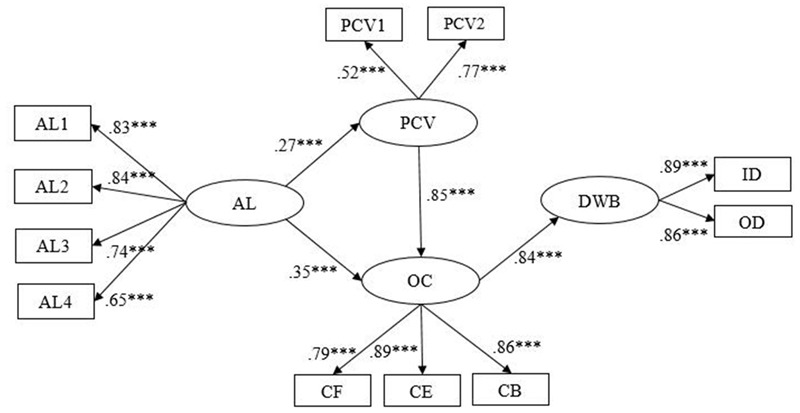
**The final mediation model (Model 3).** AL1–AL4 are four parcels of authoritarian leadership (AL1–AL3 aggregates of two items and AL4 was three items from the Authoritarian Leadership Questionnaire); PCV, psychological contract violation; OC, organizational cynicism; DWB, deviant workplace behaviors; PCV1 aggregates of two items and PCV2 was two items from Psychological Contract Violation Questionnaire; CF, cynicism faith; CB, cynicism behavior; CE, cynicism emotion; CF, CB, and CE are three dimensions of the Organizational Cynicism Questionnaire; ID, interpersonal deviation; OD, organizational deviation; ID and OD are two dimensions of the Deviant Workplace Behaviors Questionnaire. *^∗^p* < 0.05; *^∗∗^p* < 0.01; *^∗∗∗^p* < 0.001.

Second, in order to determine the best model, we developed another alternative model (Model 3). For Model 3, we deleted the non-significant path and added a path between psychological contract violation and organizational cynicism based on the Model 2 (see **Figure 4**). The chi-square difference between Model 2 and Model 3 reached the significant level (Δχ^2^ (1) = 45.84, *p* < 0.001). The results indicated that compared with Model 2, Model 3 provided a better fit to the data (see **Table 3**). Therefore, Model 3 was chosen as our final structural model.

Further, we tested the mediation effects identified in Model 3 using the bootstrapping method (Preacher and Hayes, 2008; Hayes, 2013). The SEM results supported our hypotheses. First, as depicted in **Table 4** and **Figure 4**, psychological contract violation and organizational cynicism mediated the relationship between authoritarian leadership and DWB, and the significant mediation effects comprised: (a) the indirect effect of authoritarian leadership on DWB via organizational cynicism, (b) the indirect effect of authoritarian leadership on DWB via psychological contract violation followed by organizational cynicism. Therefore, H1, H2c, and H3c were supported. Second, the path coefficient from authoritarian leadership to psychological contract violation was positively significant (β = 0.27, *p* < 0.001), supporting H2a. Third, the path coefficient between authoritarian leadership and organizational cynicism was significant (β = 0.35, *p* < 0.001), so H3a was supported. Fourth, the path coefficient from organizational cynicism to DWB was significant (*β* = 0.84, *p* < 0.001), supporting H3b. Fifth, H4 was supported by the results: the path coefficient between psychological contract violation and organizational cynicism was significant (β = 0.85, *p* < 0.001). Sixth, the indirect effect of organizational cynicism in the link between psychological contract violation and DWB (PCV→OC→DWB) was significant (β = 0.71, *p* < 0.001) (see **Table 4**). This result demonstrated that psychological contract violation was positively related to DWB, through the indirect effect of organizational cynicism. Therefore, H2b was supported.

**Table 4:** Direct and indirect effects and 95% confidence intervals in final model 3.

Model pathways	Estimated effect	95% CI	
		Lower bounds	Upper bounds
**Total effect**
AL→ DWB	0.48^∗∗∗^	0.36	0.59
**Direct effects**
AL→PCV	0.27^∗∗∗^	0.18	0.36
AL→ OC	0.35^∗∗∗^	0.27	0.42
PCV→ OC	0.85^∗∗∗^	0.78	0.92
OC→ DWB	0.84^∗∗∗^	0.77	0.90
**Indirect effects**
AL→OC→DWB	0.29^∗∗^	0.18	0.42
PCV→ OC→ DWB	0.71^∗∗∗^	0.62	0.80
AL→ PCV→ OC→ DWB	0.19^∗^	0.08	0.31

*AL, authoritarian leadership; OC, organizational cynicism; PCV, psychological contract violation; DWB, deviant workplace behaviors.*

*^∗^*p* < 0.05; *^∗∗^p* < 0.01; *^∗∗∗^p* < 0.001.*

## Discussion

Our results show that the relationship between authoritarian leadership and employees’ DWB is fully mediated by psychological contract violation and organizational cynicism, which indicates that Model 2 (fully mediated model) fits better than Model 1 (partially mediated model). This result suggests that employees under authoritarian leadership tend to perceive higher psychological contract violation and higher organization cynicism, both of which are positively related to their DWB. Moreover, we find the link between authoritarian leadership and DWB is sequentially mediated by psychological contract violation and organizational cynicism, which supports that Model 3 fits better than Model 2. It is indicated that when leader adopts an authoritarian approach, their followers are more likely to experience psychological contract violation. Then, this sense of psychological contract violation is positively related to followers’ perception of organizational cynicism, which can be associated with their DWB.

### Theoretical Implications

First, based on the Social Exchange Theory, our findings provide an alternative lens through which to understand the underlying mechanism of the authoritarian leadership-DWB relationship. Drawing from Social Exchange Theory (Gouldner, 1960; Blau, 1964), employees can react to the social exchange relationship, which is initiated and shaped by the leaders’ actions. In this light, this study demonstrates that authoritarian leadership has a positive relationship with employees’ DWB, which is consistent with previous studies (e.g., Farh and Cheng, 2000; Schuh et al., 2013). More importantly, psychological contract violation (Brown and Moshavi, 2002) and organizational cynicism (Neves, 2012), both of which could reflect the quality of social relation between leaders and followers, are found to mediate the link between authoritarian leadership and DWB. These results contribute to current research by suggesting that the propositions from Social Exchange Theory can be extended to explain the underlying mechanism behind the relationship between authoritarian leadership and followers’ behaviors.

Moreover, our work adds to the body of literature on authoritarian leadership. Overall, this study offers an important contribution to the authoritarian leadership literature by demonstrating its relationship with employees’ negative perception and behaviors towards the organization. Prior research has emphasized that authoritarian leadership has a negative relationship with subordinates’ positive attitudes and behaviors, such as organizational commitment (Erben and Güneşer, 2008) and organizational citizenship behavior (Salam et al., 1996). However, little attention has been paid to the link between authoritarian leadership and employee’s negative attitudes and behaviors, which are frequently observed in the workplace. Thus, this study enriches the existing research by demonstrating the relationship between authoritarian leadership and negative aspects (i.e., psychological contract violation, organizational cynicism and DWB).

Last but not the least, our research has advanced the understanding of the mediating roles of psychological contract violation and organizational cynicism. To the best of our knowledge, this is the first study to propose the mediating roles of both psychological contract violation and organizational cynicism within the same structural model. Previous research has only investigated the mediating role of psychological contract violation (e.g., Ahmed and Muchiri, 2014; Liao et al., in press) or organizational cynicism (e.g., Gkorezis et al., 2015) in the link between leadership and work-related aspects. In contrast to prior work, by taking both psychological contract violation and organizational cynicism into consideration, the current research has unveiled the underlying mechanism that explains the authoritarian leadership-DWB link.

### Practical Implications

Our findings hold several important managerial implications. First and foremost, our results demonstrate that authoritarian leadership is associated with a number of negative work-related aspects. In this light, the occurrence of authoritarian leadership should be reduced by carefully selecting and training supervisors. For example, the personality characteristic of “dominance” (i.e., the levels of assertiveness, aggression, and cooperation) assessed by 16PF Questionnaire (Cattell et al., 1970) should be considered as one of indices in the supervisor recruitment system. Moreover, organizations might provide supervisors with training courses to improve their interpersonal relationship skills (Aryee et al., 2007). Additionally, leaders themselves should try to create an equity and harmonious working environment, and show benevolence to employees. However, it is noteworthy that the relationship between authoritarian leadership and poor performance of subordinates might not always be stable (Farh et al., 2008). Under some circumstances, such as tight deadlines and crisis situations, authoritarian leadership may be needed to obtain desirable outcomes. For example, authoritarian leadership could be effective in promoting subordinates’ effort in urgent situation (Niu et al., 2009), and giving them motivation to achieve goals (Chan et al., 2013). Thus, it is suggested that leaders should apply different leadership style under different situations (Yang et al., 2012). In other words, the overuse of any one leadership style may disappoint employees or harm leadership effectiveness.

Moreover, in line with previous studies, our results have demonstrated that psychological contract violation is positively related to negative attitude of cynicism (e.g., Chrobot-Mason, 2003; Johnson and O’Leary-Kelly, 2003), and employees’ DWB (e.g., Restubog et al., 2007; Bordia et al., 2008). Thus, leaders should adopt some management strategies to fulfill followers’ psychological contract. For example, leaders can ask for subordinates’ opinions to elevate their sense of participation, share management power to inspire employees’ loyalty to the organization, and show concern for subordinates’ well-being in both work and family domains.

Finally, in order to reduce organizational cynicism in the workplace, organization should use different approaches to increase trustworthiness, such as offering organizational support, treating all the employees fairly. In addition, organizations should provide a communication platform where employees could share work-related information, keep track of the company policies, and complain online with supervisors, so that a better exchange relationship would be built based on trust.

### Limitations and Future Research

The results of this research should be interpreted with respect to a number of limitations that may shed light on future research directions. First, this research is cross-sectional in nature, and no causal relationship between variables of interest in our study could be established. The longitudinal effects of organizational or psychological factors on employees’ DWB remain unexplored. It is possible that a one-occasion test of a mediation model is not adequate, particularly as a temporal sequence is proposed in our study. Thus, addressing the causality issue using a longitudinal design to test the current study model would nevertheless be a fruitful avenue for future research.

Second, although our research has demonstrated that authoritarian leadership can be positively linked with employees’ DWB. However, it is also found that if leaders perceive that the goals of organization or their own interests are defeated by followers’ behaviors, they would implement a destructive leadership style (Krasikova et al., 2013). It is suggested that followers’ DWB might be predictive of leaders’ behaviors, such as authoritarian leadership. As mentioned before, leaders may vary behaviors according to different circumstances (Yang et al., 2012). Therefore, it is recommended that future research should examine the role of followers in shaping leaders’ behaviors.

Third, this study only focuses on psychological contract violation and organizational cynicism as mediating variables. Testing other mediators such as perceived organizational support and leader-member exchange, may provide further insights regarding the mechanism underlying the link between authoritarian leadership and employees’ DWB.

Despite these limitations, this study makes several important contributions. To our knowledge, the current research represents the first attempt to investigate both psychological contract violation and organizational cynicism in one study to examine the underlying mechanism behind the link between authoritarian leadership and employees’ DWB. The results suggest that the relationship between authoritarian leadership and DWB is mediated by organizational cynicism. Moreover, this relationship is also sequentially mediated by psychological contract violation and organizational cynicism. In consideration of the probable mechanism, these findings could provide valuable guidance for how to reduce employees’ DWB.

## Ethics Statement

This study was carried out in accordance with the recommendations of ethics committee of China University of Mining and Technology with written informed consent from all subjects. All subjects gave written informed consent in accordance with the Declaration of Helsinki. The protocol was approved by the ethics committee of China University of Mining and Technology.

## Author Contributions

HJ: substantial contributions to the conception and design of the work, drafting and revising the paper, and agreement to be accountable for all aspects of the work in ensuring that questions related to the accuracy of any part of the work are appropriately investigated and resolved. YC: substantial contributions to drafting the work and revising it critically for important intellectual content. PS: substantial contributions to the conception and design of the work, and the communication with the journal during the manuscript submission, peer review, and publication process. JY: substantial contributions to data acquisition and data analysis.

## Conflict of Interest Statement

The authors declare that the research was conducted in the absence of any commercial or financial relationships that could be construed as a potential conflict of interest.
